# Experimental and numerical evaluations of composite concrete-to-concrete interfacial shear strength under horizontal and normal stresses

**DOI:** 10.1371/journal.pone.0252050

**Published:** 2021-05-20

**Authors:** M. Yahya Al-Fasih, M. E. Mohamad, I. S. Ibrahim, Y. Ahmad, M. A. Mohd Ariffin, N. N. Sarbini, R. N. Mohamed, A. B. H. Kueh

**Affiliations:** 1 Faculty of Engineering, Forensic Engineering Centre (FEC), Institute of Smart Infrastructure and Innovative Construction (ISIIC), School of Civil Engineering, Universiti Teknologi Malaysia, Johor Bahru, Johor, Malaysia; 2 Office of Education, Sana’a, Yemen; 3 School of Engineering and Technology, University College of Technology Sarawak, Sibu, Sarawak, Malaysia; 4 Faculty of Engineering, School of Civil Engineering, Universiti Teknologi Malaysia, Johor Bahru, Johor, Malaysia; 5 Faculty of Engineering, Department of Civil Engineering, Universiti Malaysia Sarawak, Kota Samarahan, Sarawak, Malaysia; China University of Mining and Technology, CHINA

## Abstract

Effects of different surface textures on the interface shear strength, interface slip, and failure modes of the concrete-to-concrete bond are examined through finite element numerical model and experimental methods in the presence of the horizontal load with ‘push-off’ technique under different normal stresses. Three different surface textures are considered; smooth, indented, and transversely roughened to finish the top surfaces of the concrete bases. In the three-dimensional modeling via the ABAQUS solver, the Cohesive Zone Model (CZM) is used to simulate the interface shear failure. It is observed that the interface shear strength increases with the applied normal stress. The transversely roughened surface achieves the highest interface shear strength compared with those finished with the indented and smooth approaches. The smooth and indented surfaces are controlled by the adhesive failure mode while the transversely roughened surface is dominated by the cohesive failure mode. Also, it is observed that the CZM approach can accurately model the interface shear failure with 3–29% differences between the modeled and the experimental test findings.

## Introduction

The composite concrete slab is constructed by integrating a precast concrete slab with the cast-in-place concrete topping, both of which are performing monolithically in unison as a single element as dictated by their shear strength at the interface. The interfacial behavior of composite concrete slab plays an imperative role in maintaining its integrity in service. In essence, the cohesion and frictional characteristics of concrete, as well as its external normal stress all contribute to the capacity of the composite action near the interface region of the slab [1–3]. The shear-friction theory advocates that the shear is transmitted across the interface between two concrete members along with the occurrence of friction-inflicted normal forces acting on the interface [4–6]. In the interface of a composite slab, failure may occur in the concrete topping, which tends to not conform to the curvature of the precast slab under a deformed state [7]. Therefore, understanding the interface texture behaviors of the concrete-to-concrete composite may contribute to an efficient interface shear strength design of the slab under horizontal and normal loadings.

A significant number of experimental studies have revealed that the interface shear strength relies greatly on the roughness and the adhesive type of the composite interface. Loov and Patnaik [8] pointed out that an effective interface shear strength is critical for the development of flexural strength, shear strength, and deflection characteristics. Through a series of investigations, Mohamad *et al*. [9], Ceia *et al*. [10], and He *et al*. [11] observed that the shear strength of the concrete-to-concrete interface increases with its roughness. Costa *et al*. [12] stated that the binding matrix strength and the type of aggregate influence the interface strength attributed to its dependence on the roughness of the substrates. By investigating the influence of carbonation on the shear strength prediction between the old concrete substrate and the new self-compacting concrete (SCC) overlay, Zhang [13] showed that the shear strength increases by 30% when the carbonation depth of the substrate is deeper than 20 mm.

Over the past decades, various techniques have been established to model the fracture or debonding of the interface layers through the fracture mechanics and strength of materials methods [14–17]. As an alternative, the Cohesive Zone Model (CZM) pioneered by Dugda in 1960 [18] and Barenblatt in 1962 [19] is a fracture mechanics model that directly introduces the fracture mechanism by adopting the softening relationship between traction and separation in numerical simulations [20,21]. They had been successfully used CZM to simulate and predict the entire fracture process from crack onset to rupture, including crack growth, propagation, potential bifurcation, and multiple fracturing. Alfano [22] presented the effects of the shape of the interface law (e.g., bilinear, linear-parabolic, exponential, and trapezoidal) on the analysis of debonding by using CZM. They found that the exponential law has the optimal finite-element approximation and bilinear law has the best agreement between the approximation and compactional cost while the trapezoidal law has worst results in term of numerical stability. Borst *et al*. [23] implemented CZM to simulate the crack propagation by considering the partition-of-unity property of the finite element mesh to avoid any mesh bias. The fracture of quasi-brittle materials such as concrete was defined using CZM for the concrete-to-concrete behavioral simulation technique [24]. Hadjazi *et al*. [25] established theoretical model based on the bi-linear CZM for intermediate crack-introduced debonding in FRP-plated concrete beam. They found that the capacity of the FRP-concrete interface increased with the increase of crack length and the thickness of the FRP plate. Additionally, a cohesive or bridging zone model was developed to simulate the interfacial debonding between fiber-reinforced polymer (FRP) and concrete with the consideration of different failure mechanisms at the bridging zone and softening zone [26]. The model confirmed that the pulling force on the FRP plate is directly associated with the square root of the energy release rate at the debonding tip.

**Fig 1:**
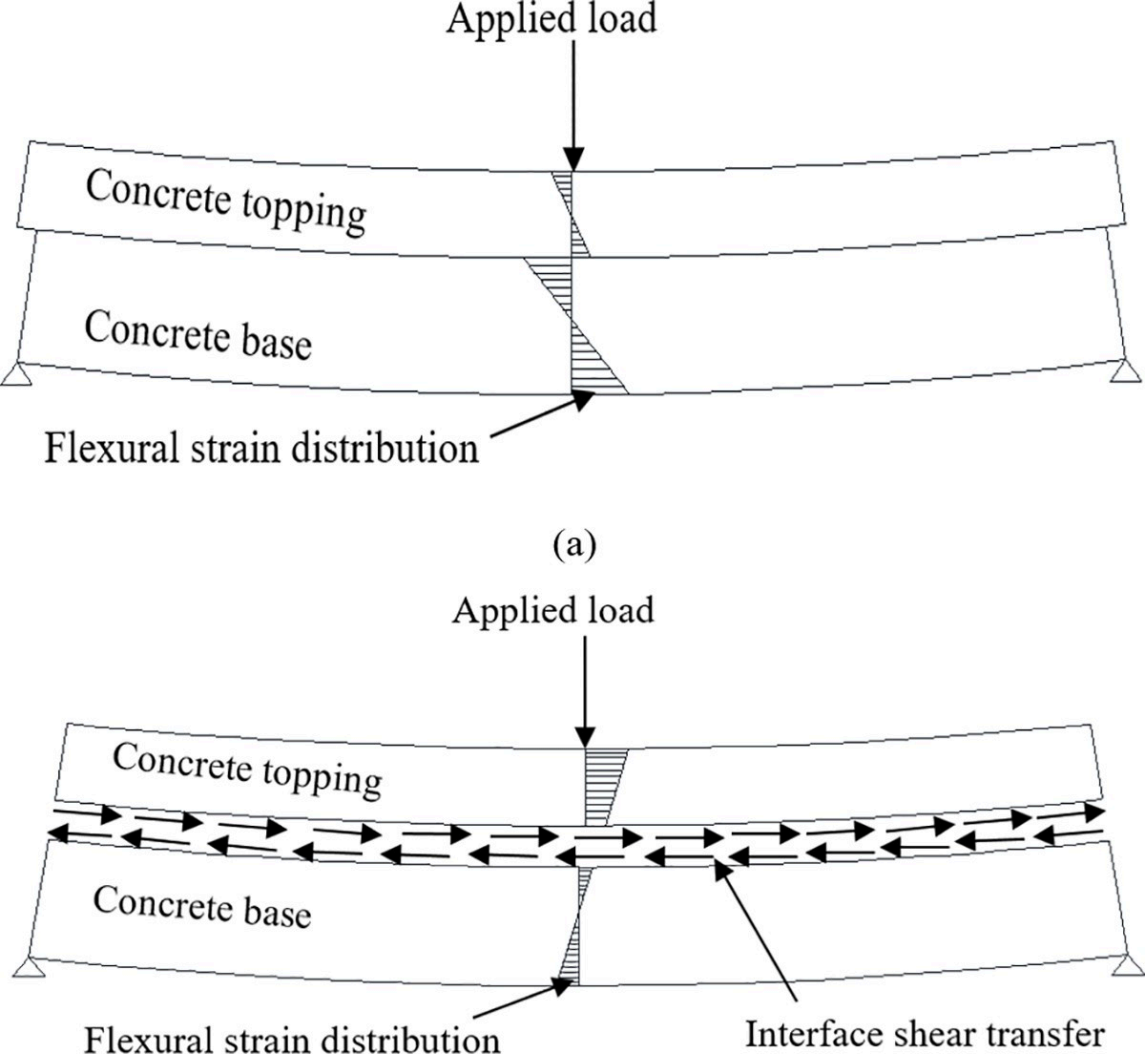
Stress distribution of a) Non-composite section and b) Composite section (Kovach and Naito [27]).

It is worth noting that most of the aforementioned experimental studies considered only the horizontal loading case. However, in reality, the vertical load occurs also on the top layer of the composite precast slab during service. For the composite slab to behave monolithically, the bond at the interface between the precast slab and concrete topping must remain intact. The interface shear stress must be sufficiently transferred along the interface of the two concretes. However, when load is applied on the weaker interface bond, it may cause interface failure due to slippage of the concrete topping. If this slip occurred and the composite action is lost, only friction force is acted between the precast slab and concrete topping. Therefore, each concrete layer will deform separately due to the vertical forces which cause tension at the bottom of the two concretes. Fig 1 shows the stress distributions for the weak interface bond (non-composite section) and the strong interface bond (composite section) of the composite precast concrete slab behavior. In terms of simulation, finite element modeling has not been performed for the concrete-to-concrete composite through the “push-off” test with the consideration of simultaneous effects of both horizontal and normal loads.

Therefore, this study was aimed to investigate the effects of the surface texture roughness on the interface shear strength, interface slip, and failure mode of the interface of the concrete-to-concrete composite under both horizontal and vertical loading using the “push-off” test method. To evaluate the quality of the surface which ranges from very smooth to very rough (limited to the roughness height) and is significantly influenced by the method of preparation as per Eurocode 2 [28], smooth or “left as-cast” with a troweled finish, the indented surface finished with a corrugated steel mold, and the transverse roughened surface finished by wire-brushing in the transverse direction surfaces were considered and measured with the aid of a roughness instrument. Also, the finite element (FE) numerical modeling was employed with the ABAQUS/Standard [29] solver to predict the interface strength between the concrete base and topping layers, adopting specifically the Cohesive Zone Model (CZM) interaction description. Conforming to the physical findings, the results from the experimental tests were implemented in the model to simulate the interface bond. Furthermore, outcomes from the horizontal load, interface slip, and interface shear strength from both numerical and experimental approaches for the “push-off” tests were compared for verification purposes. Departing from a successful verification, the models were then employed to examine the stress pattern and failure mode experienced by different conditions of surface textures at the interface region. The paper ends by highlighting the main findings from the current study.

### Experimental program

**Fig 2:**
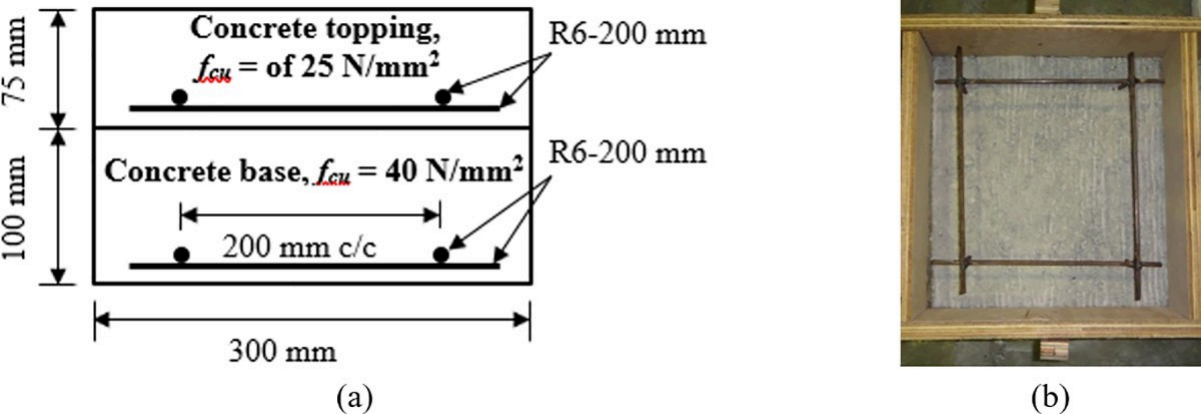
“Push-off” specimen a) Schematic of reinforcement details, and b) Actual reinforcement.

The “push-off” test used to study the behavior of the interface bond of the composite concrete-to-concrete slab under different normal stresses. The test was conducted following the approach adopted in Mohamad *et al*. [9]. A total of 36 (12 specimens for each surface texture, i.e., 3 specimens for each normal stress) were prepared. The overall dimension of the specimen is 300 mm × 300 mm × 175 mm. The concrete base as the bottom layer is 100 mm high while the concrete topping as the top layer is 75 mm high. Mild steel wire mesh of 6 mm diameter and at 200 mm spacing were used in both concrete base and concrete topping to control shrinkage as shown in Fig 2. These concrete layers were, respectively, designed with compressive strengths of 25 N/mm^2^ and 40 N/mm^2^. In preparation, the concrete topping was cast onto the concrete base after confirming that the concrete base had achieved two-third of the design compressive strength at 7 days.

**Fig 3:**
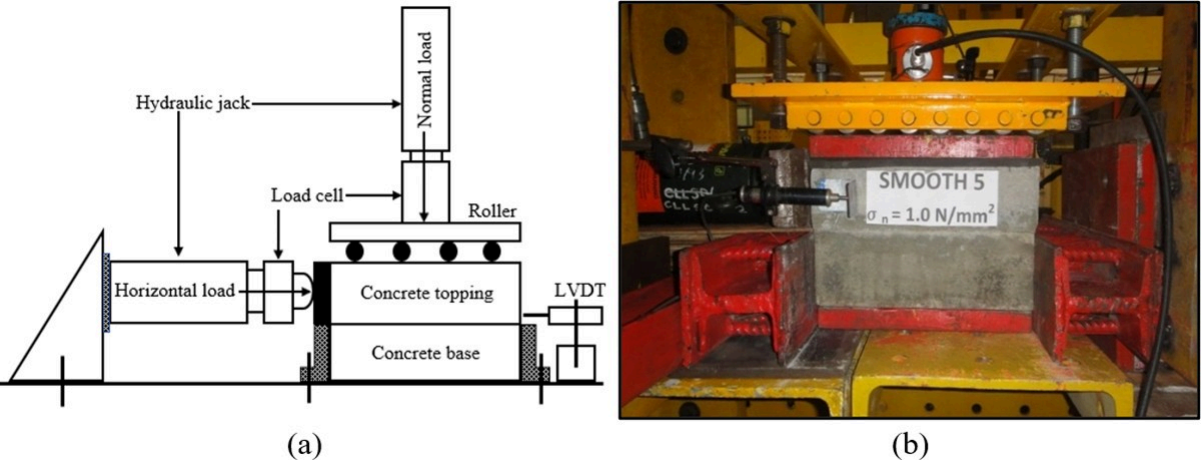
“Push-off” test setup: a) Schematic drawing, and b) Experimental set-up.

Fig 3 illustrates the “push-off” test set up. A horizontal load parallel to one of the interface bond lines was applied to one side of the concrete topping while the concrete base was fixed to the test frame to attain a complete shear failure at the interfacial region. Also, four normal stresses were considered: 0 N/mm^2^, 0.5 N/mm^2^, 1.0 N/mm^2^, and 1.5 N/mm^2^. To avoid any uplift during the test, a uniformly arranged set of rollers was placed on top of the specimen. The horizontal displacement or interface slip was measured during the test using the Linear Variable Displacement Transducer (LVDT) close to the interface as shown in Fig 3. The horizontal load is applied incrementally at every 5 kN until the specimen failed. Failure is well defined when the bond at the interface is broken or when the two concrete layers become separated.

**Fig 4:**
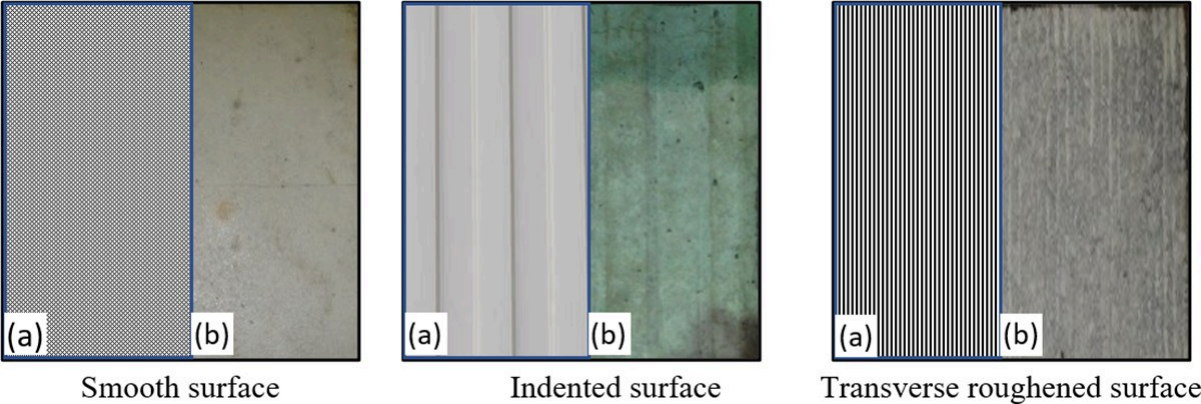
Surface textures of the top surface of the concrete base: a) schematics and b) actual surfaces.

**Fig 5:**
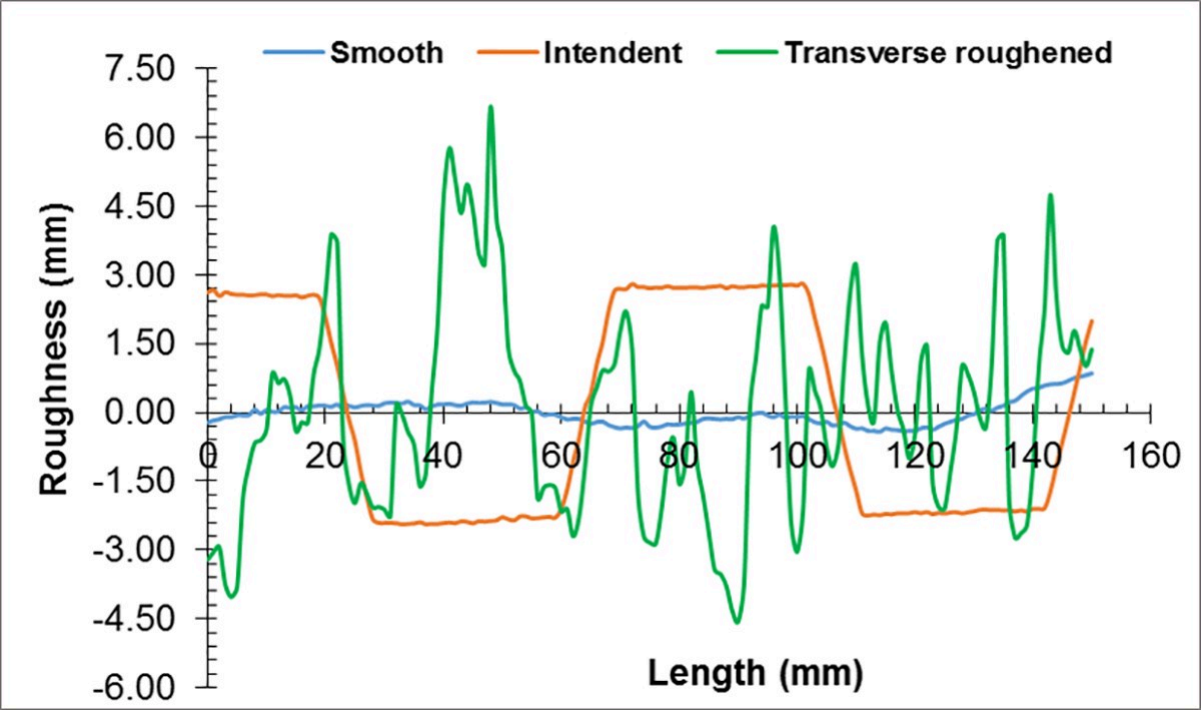
Surface roughness profile at the top surface of the concrete base.

Fig 4 shows the surface textures at the top face of the concrete base while Fig 5 displays their surface roughness profiles as assessed by the Portable Stylus roughness instrument. Three different surface textures were examined, including smooth, indented, and transversely roughened. These surfaces were left as-cast with a troweled finishing, with a corrugated steel mold, and with wire-brushing in the transverse direction, respectively.

The interface shear strength was determined based on the shear-friction theory for the surface texture types examined in this study as follows [9]:

$$\tau =C.f_{ct}+\mu .\sigma_{n}$$ (1)

where *f*_*ct*_ is the lower concrete tensile strength among the two layers, *σ*_*n*_ is the normal stress strength, and *μ* is the friction coefficient compatible with the interface shear strength given as:

$$\mu =0.8766\;{R_{pm}}^{0.3978}$$ (2)

where *R*_*pm*_ is the mean peak height roughness parameter while the theoretical cohesion coefficient, *C*, takes the following expression:

$$C=0.2363e^{0.237R_{pm}}$$ (3)

### Finite element numerical modeling

#### Modeling description

**Fig 6:**
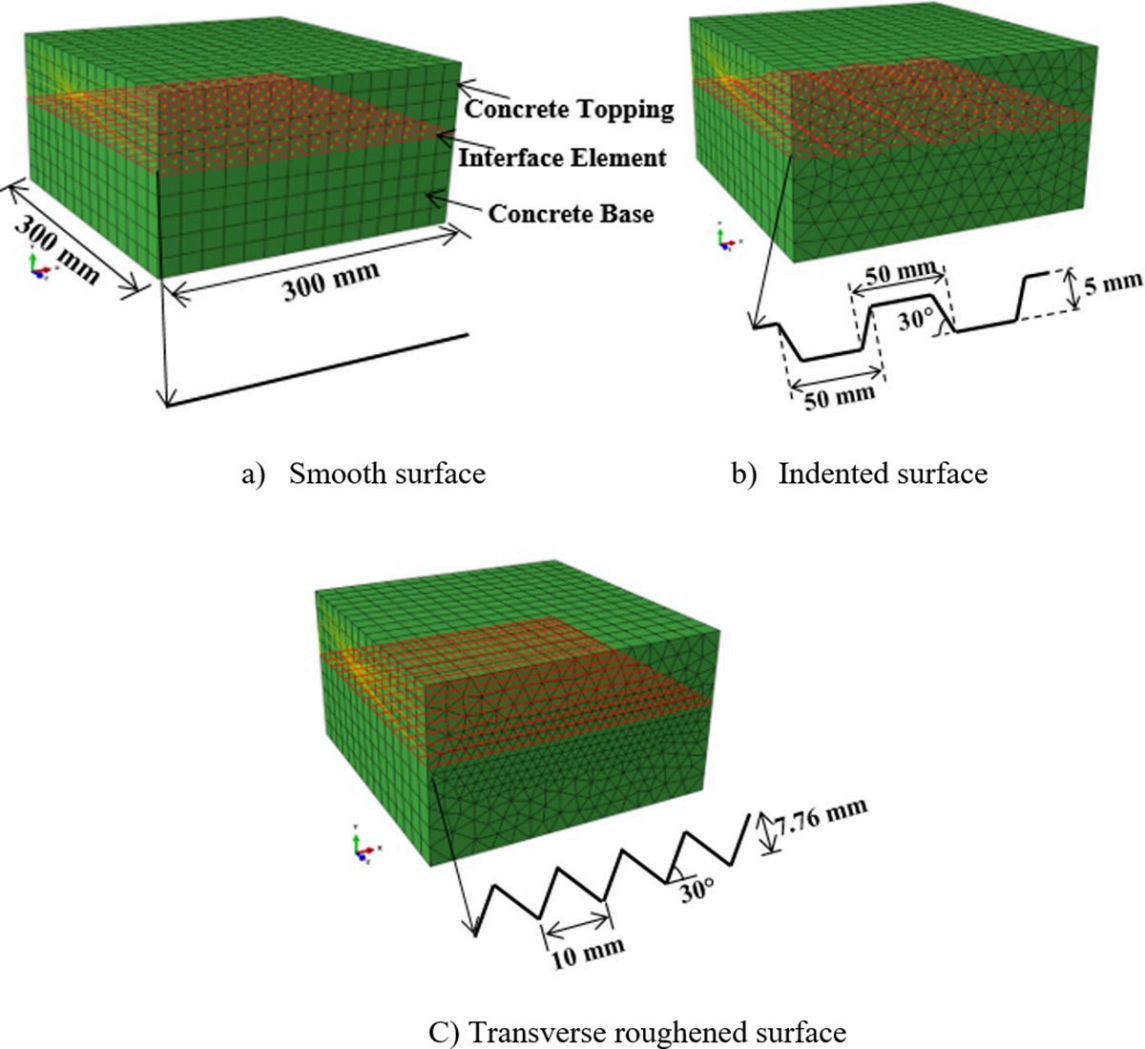
FE models for the composite concrete-to-concrete specimens with various interface types.

In addition to the physical tests, the “push-off” tests were simulated by the 3D FE models using the commercial software, ABAQUS 6.12/standard [29]. Fig 6 shows the modeled composite concrete-to-concrete specimens with the considered three interface textures, i.e., smooth, indented, and transversely roughened. All dimensions and parameters of the interface were modeled identically with the experimental specimens.

**Fig 7:**
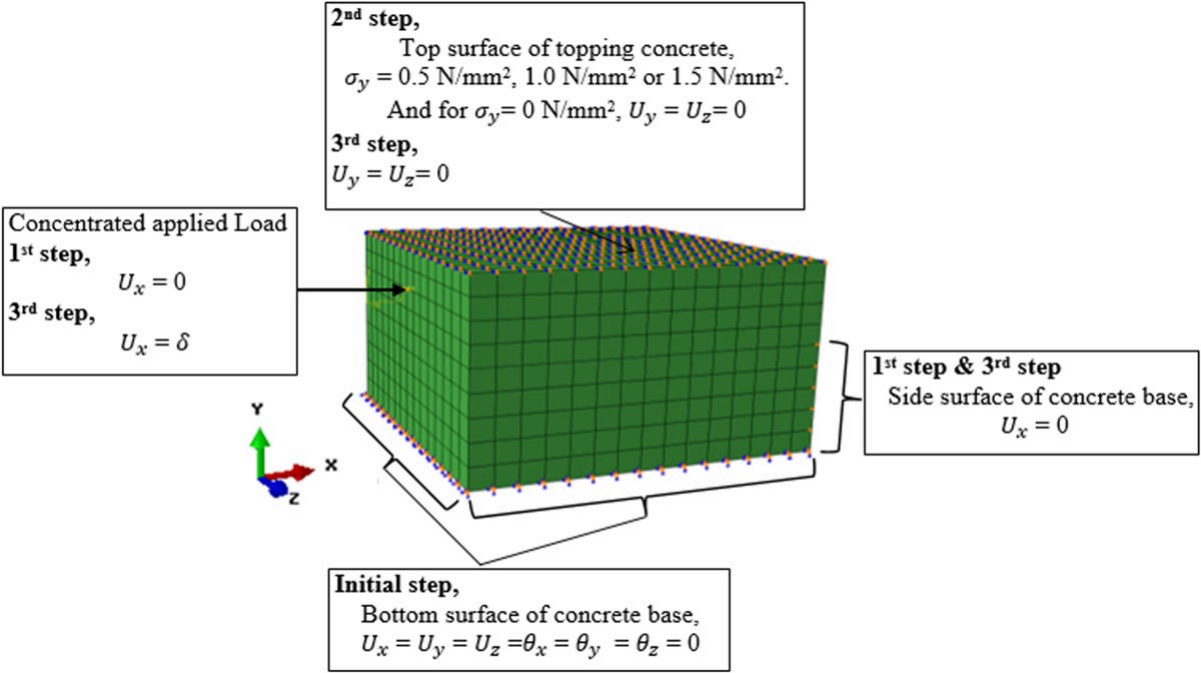
Evolution of the boundary conditions to simulate the loading sequence.

The interaction characteristics of the interface element introduced between the concrete base and concrete topping were modeled in accordance with the interface failure modes demonstrated in the experimental tests. Accordingly, the interaction of the interface element was modeled adopting the tie constraint description for the smooth and indented surfaces due to the observed adhesive bond failure. On the other hand, for the transversely roughened surface, the interface failed with the cohesive mode. To model such a condition, the interface element was inserted through the concrete topping. Meanwhile, the rough interface for the concrete-to-concrete bond was modeled as the cohesive interaction instead of an element-based interfacial description (see Fig 6). To perform the load-interface slip analysis along with the normal stress, the boundary conditions were applied in the FE modeling step-by-step as illustrated in Fig 7. In the initial step, the bottom surface of the model was constrained in all directions. In the first step, both the side surfaces of the concrete topping and concrete base were restrained. In the second step, normal stress was applied onto the top surface of the model at 0.5 N/mm^2^, 1.0 N/mm^2^, and 1.5 N/mm^2^ for different normal stress loading conditions. For the control specimen where the normal stress was 0 N/mm^2^, the top surface was restrained in the *y*- and *z*-directions. In the third step, the load was applied as a displacement, *δ*, monotonically in the *x*-direction until the interface slipped and the loading curve experienced a drop in magnitude. The application of loading on the composite concrete used the kinematic coupling constraint, in which a large number of nodes (also known as the “coupling” nodes) were constrained to the rigid body motion of a single reference point located centrally at the side of the concrete topping.

The concrete base and concrete topping were modeled as elastic materials with Young’s Moduli of 35 kN/mm^2^ and 31 kN/mm^2^, respectively, as obtained from the experimental tests. The concrete base and concrete topping models were meshed using the 3-dimensional solid elements. The element type employed for the smooth surface specimen was an 8-node linear bricks (hexahedral) with reduced integration and hourglass control (C3D8R) while those with indented and transversely roughened surfaces were meshed with 6-node linear triangular prism elements (C3D6). The selection of different types of continuum elements was due to differences in the surface textures. The 3-dimensional interface element (COH3D8) with zero-thickness was embedded in the model via shared nodes or tie constraints to connect the concrete topping and concrete base.

The CZM parameters were then used to describe the interface shear behavior of the models. The employed CZM included the constitutive relation between the traction acting on the interface and the corresponding interface separation (displacement or slip at the interface). The traction-separation law was applied by assuming the isotropic condition in the fracture energy, in which *G*_*IC*_ = *G*_*IIC*_ = *G*_*IIIC*_ = *G*_*TC*_. The critical fracture energy (*G*_*TC*_) was determined as:

$$G_{TC}=\frac{1}{2}\times S\times \delta$$ (4)

where *S* is the peak shear load and *δ* is the displacement at failure.

The elastic modulus of the traction-separation law was obtained as the initial slope that relates the interface shear strength to the displacement given as:

$$K_{s}=\frac{S_{max}}{\delta_{s}^{init}}$$ (5)

The onset interface shear failure takes place when the corresponding interface traction exceeds its maximum interface shear strength. The model interface shear strength was considered as isotropic. The damage was assumed to initiate when the quadratic interaction function containing the interface shear stress (nominal stress) ratios reaches 1.0. The relevant expression is:

$${\left\{\frac{t_{n}}{t_{n}^{0}}\right\}}^{2}+{\left\{\frac{t_{s}}{t_{s}^{0}}\right\}}^{2}+{\left\{\frac{t_{t}}{\tau_{t}^{0}}\right\}}^{2}=1$$ (6)

where *t*_*n*_ is the normal traction, *t*_*s*_ and *t*_*t*_ are the transverse tractions, and $t_{n}^{0},\;t_{s}^{0}$, and $t_{t}^{0}$ are the nominal tensile and shear strengths, respectively.

In the analysis, the energy-based Benzeggagh-Kenane (BK) damage evolution criterion given as:

$${G_{TC}=G}_{IC}+(G_{IIC}-G_{IC}){\left\{\frac{G_{shear}}{G_{T}}\right\}}^{\eta }$$ (7)

was adopted where *G*_*shear*_ = *G*_*II*_+*G*_*III*_, *G*_*T*_ = *G*_*I*_+*G*_*shear*_, and *η* is the BK material parameter. For isotropic failure, *G*_*IC*_ = *G*_*IIC*_ and *η* was taken as 1.

The obtained interface properties for the composite concrete are summarized in Table 1. The interface shear strength and fracture energy are the parameters that control the peak load of the concrete interface, while the elastic shear stiffness replicates the linear elastic pattern found in the experimental results.

**Table 1:** Interface properties for the composite concrete.

Surface Texture	Normal Stress (N/mm^2^)	Interface Shear Strength (N/mm^2^)	Elastic Shear Stiffness (N/mm^2^)	Fracture Energy (N/mm)
Smooth	0	0.73	0.48	1.37
	0.5	1.51	0.98	2.80
	1.0	1.81	0.75	4.12
	1.5	2.11	2.02	2.63
Indented	0	1.55	0.70	5.68
	0.5	2.55	0.87	5.54
	1.0	3.22	0.82	9.78
	1.5	3.72	1.14	15.83
Transversely roughened	0	3.78	0.66	25.54
	0.5	4.84	1.03	22.14
	1.0	6.00	1.38	21.25
	1.5	6.51	1.54	26.10

The refinement meshes for monolithic plain concrete were carried out for all the surface textures with the element size of 5 mm, 15 mm, and 20 mm. From the converged outcome, an average element size of 20 mm was considered in all the models. The employed numbers of nodes and elements for each model are summarized in Table 2.

**Table 2:** Finite element mesh densities for all models.

Surface Texture	Size (mm)	Total Number of Elements		
		Node	Continuum Element	Interface Element
Smooth	5	141398	126000	3600
	15	6615	4800	400
	20	3072	2025	225
Indented	5	147681	263880	3720
	15	7917	11760	480
	20	4000	5490	300
Transverse	5	157746	276960	3600
	15	11718	17320	600
	20	7296	10620	450

The horizontal load-interface slip relationship was plotted for each normal stress loading condition. The interface shear strength was determined from the average maximum value of all nodes on the interface layer.

## Experimental results

### Horizontal load-interface slip relationship

**Fig 8:**
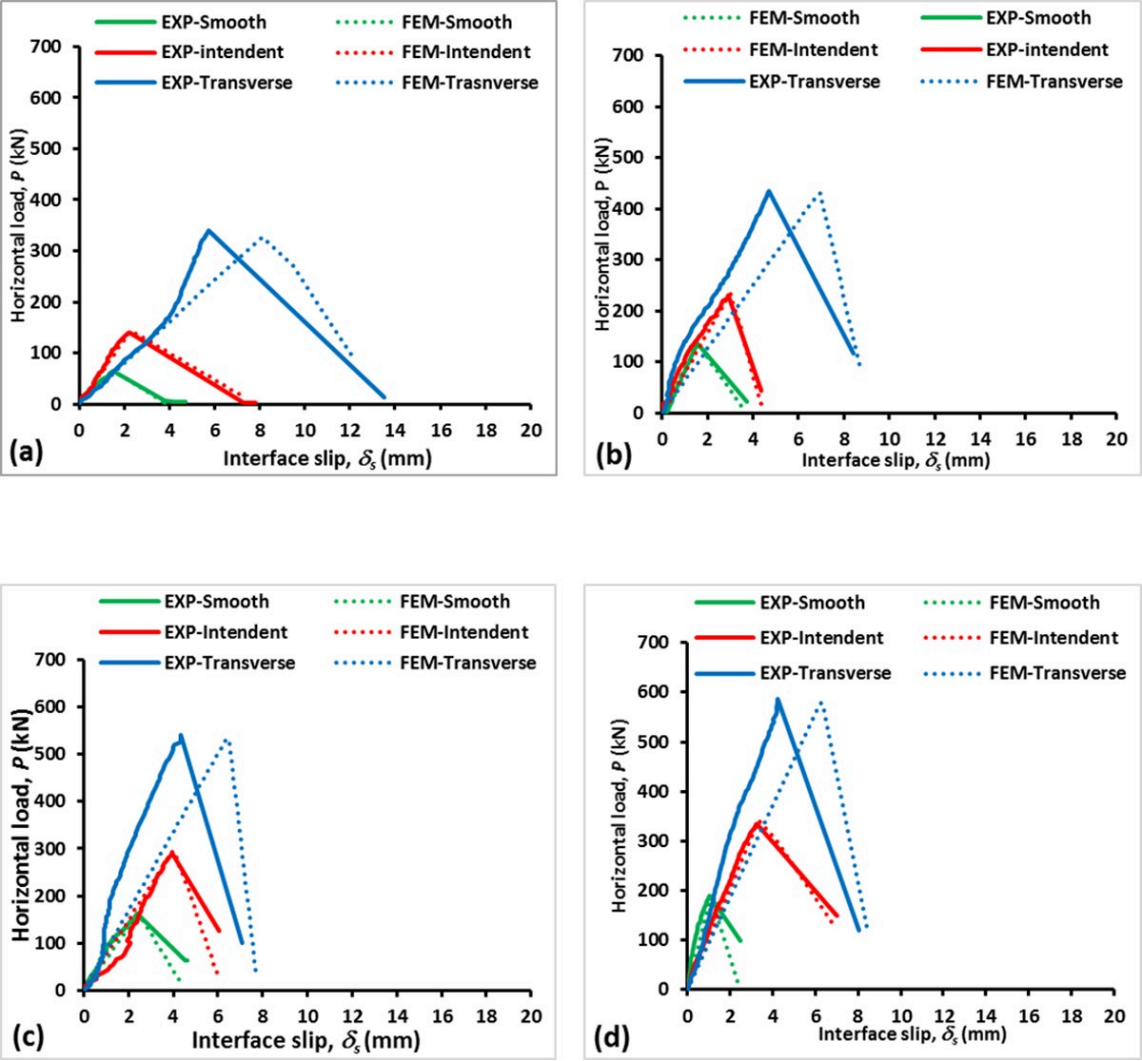
Horizontal load–interface slip relationship under various normal stresses: *σ*_*n*_ = (a) 0 N/mm^2^, (b) 0.5 N/mm^2^, (c) 1.0 N/mm^2^, and (d) 1.5 N/mm^2^.

The relationships of horizontal load and interface slip in the presence of normal stresses for different surface textures are shown in Fig 8. It can be noticed that the horizontal load increases linearly with interface slip until failure. The failure load is described as the peak shear load before the breakage of the cohesion bond at the interface [30]. The failure load increases corresponding to the rise in the normal stress from 0 N/mm^2^ to 1.5 N/mm^2^. At the attainment of the failure load, slipping was developed and eventually resulted in the separation from the two concrete layers.

**Fig 9:**
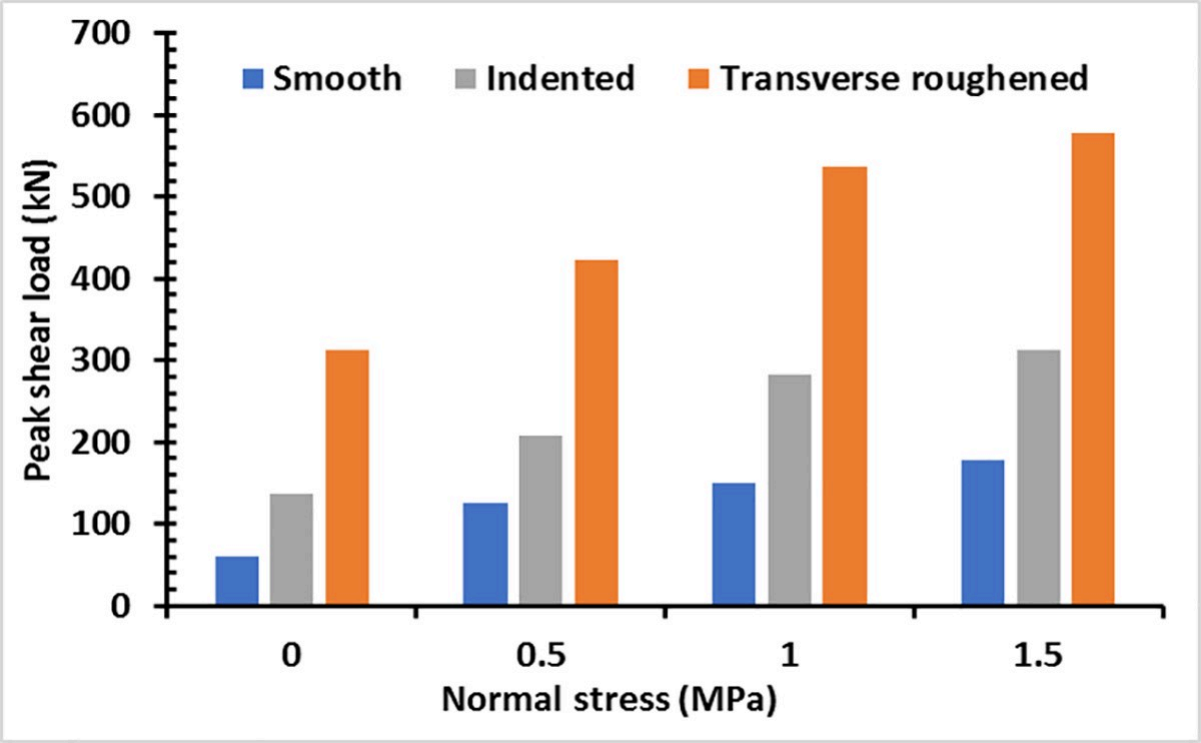
Peak shear loads of various interfacial conditions under different normal stresses.

After the peak shear load was reached, a linear drop trend can be observed in the load magnitude following the increment of the interface slip, indicating the beginning of interface bond failure. The corresponding lowest load magnitudes were close to 0 kN for all the specimens except in some cases depending on the applied normal stress. The state at which the peak load is attained before the concrete layers are separated is referred to as the pre-crack interface shear strength. During the early loading process, both the interface slip and the horizontal load increased gradually under the static friction condition. In this stage, the horizontal load broke the interface bond by exceeding the pre-crack interface shear strength. The transversely roughened surface specimens recorded the greatest peak shear loads ranging from 311.77 kN to 577.30 kN for all normal stress conditions before the interface bond was broken. This was followed by the specimens with the indented surfaces and then those with smooth surfaces with peak shear loads between 136.73 kN and 335 kN as well as between 60.3 kN and 178.3 kN, respectively, as exhibited in Fig 9. This implies that although the specimens with indented surfaces were cast with corrugated steel, they achieved lower bonding strength when higher compressive stress was applied compared with those with transversely roughened surfaces due to smooth surface condition in the former specimens. This confirms that the rougher surface texture produces a comparatively higher interface shear strength.

In the simulation, the damage initiation was computed at the peak shear load while the propagation was initiated at the softening portion of the curve. It can be seen in Fig 8 that the softening curve progresses in a decreased manner until it reaches a certain load due to the residual of the applied normal stress. The bi-linear relationship of the traction-separation behavior is observed as a sudden interface slip jump after reaching the maximum load. This is then followed by a gradual drop in load with a large increase in the interface slip for all models. The drop indicates that the interface is broken or damaged.

The comparative differences in the peak shear loads between the FE model and experimental test are 3.17%, 9.94%, 10.92%, and 4.69% for *σ*_*n*_ = 0 N/mm^2^, 0.5 N/mm^2^, 1.0 N/mm^2^, and 1.5 N/mm^2^, respectively. The specimen under *σ*_*n*_ = 0 N/mm^2^ shows that its corresponding horizontal load increases linearly with a clear yield. At this point, the plasticity takes place with little hardening in following the perfectly plastic properties. In the FE model, the elastic behavior of the interface layer was assumed as isotropic for the concrete topping, hence, the slight difference in stiffness between the FE model and the experimental test. However, the linear elastic behavior of the FE model at *σ*_*n*_ = 0.5 N/mm^2^ is close to the experimental test.

The load-deformation curves of transversely roughened specimens show that the peak shear loads of both the experimental test and the FE model are close to each other with the percentages of difference ranging from 1.02% to 3.66%. Meanwhile, the interface slips of the experimental specimens at peak shear load are 29.36% and 33.02% lower than the FE model. This is because the surface of the transversely roughened specimen was modeled as cohesive behavior by assuming that the failure occurred at the concrete topping. However, in the experimental test, the interface between the concrete base and concrete topping can be considered as having a full bonding.

#### Interface shear strength

Table 3 shows the resulted interface shear strengths obtained from both experimental tests (EXP) and FE models. The interface shear strengths for the experiment specimens were calculated using Eq (1) while those for the FE models were extracted from the average maximum interface shear stress from all nodes on the interface layer. 6% to 29%, 4% to 14%, and 3% to 21% differences are detected between the modeled and experimental results for smooth, indented, and transversely roughened specimens, respectively. Hence, a good agreement of the FE models with all experimentally obtained outcomes can be noticed.

**Table 3:** Interface shear strength from the experimental and modeled results.

Surface Texture	Normal Stress, *σ*_*n*_ (N/mm^2^)	Interface Shear Strength		$\frac{\tau_{FEM}}{\tau_{EXP}}$
		*τ*_*FEM*_ (N/mm^2^)	*τ*_*EXP*_ (N/mm^2^)	
Smooth	0	0.62	0.87	0.71
	0.5	1.28	1.36	0.94
	1.0	1.54	1.83	0.84
	1.5	1.78	2.40	0.74
Indented	0	1.41	1.64	0.86
	0.5	2.31	2.40	0.96
	1.0	2.91	3.10	0.94
	1.5	3.35	3.88	0.86
Transversely roughened	0	2.93	3.63	0.81
	0.5	3.87	4.91	0.79
	1.0	4.80	6.22	0.77
	1.5	5.21	5.06	1.03

### Interface failure mode

**Fig 10:**
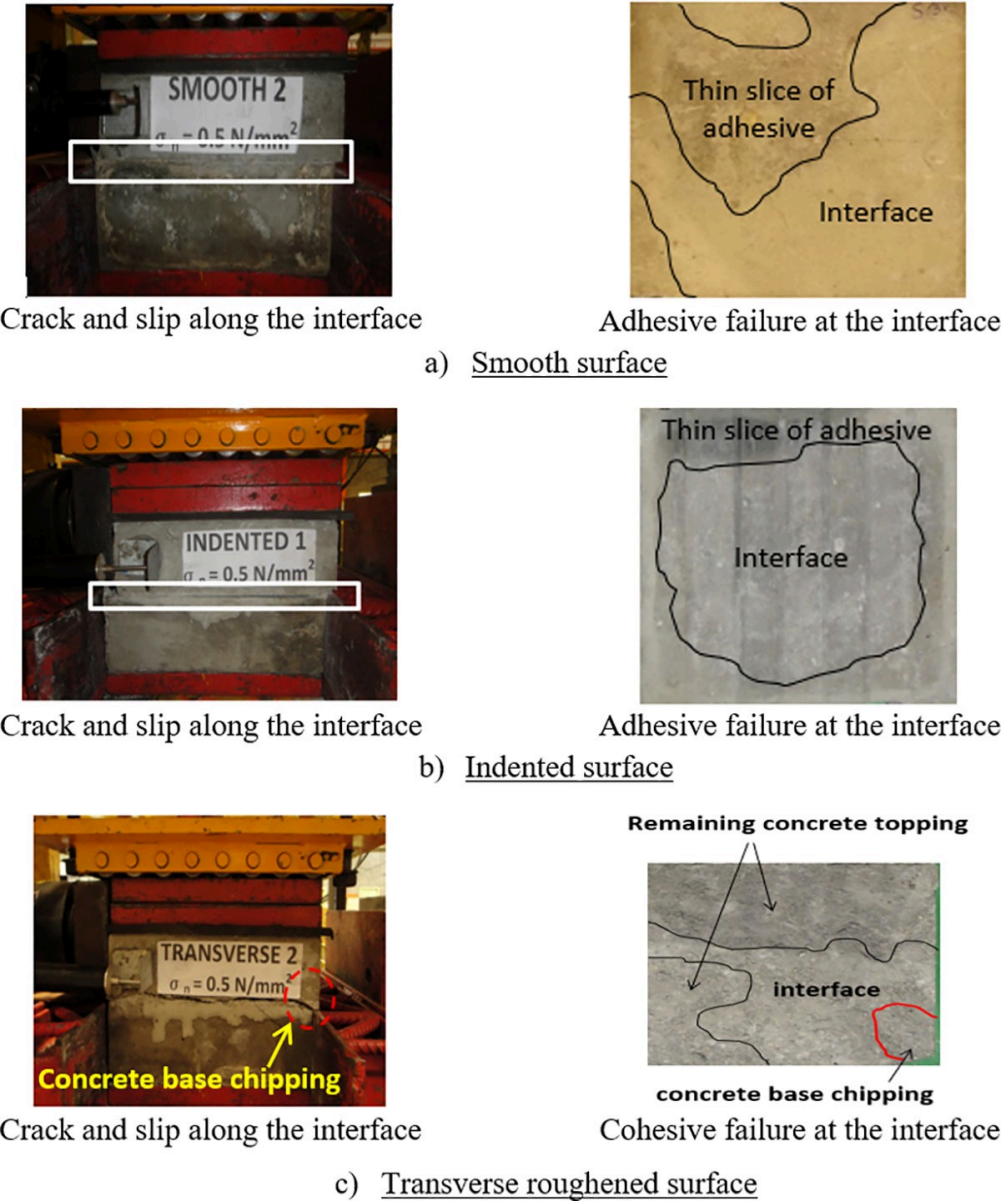
Failure mode at the interface regions.

It was observed that the composite slab failed under the simultaneous actions of normal stress and “push-off” loadings in two specific modes; adhesive and cohesive failures. The rupture of the interface bond is referred to as the adhesive failure, in which separation occurs at the interface zone while the chipping of concrete base or topping is referred to as the cohesive failure. The smooth and indented specimens failed by the adhesive mode where a thin slice of concrete topping adhesive could be detected on the smooth and indented surfaces as depicted in Fig 10(A) and 10(B). However, the transversely roughened specimens failed by the cohesive mode where the failure occurred partially along the interface bond and in the concrete topping as shown in Fig 10(C). Furthermore, chipping was observed at the concrete base as small pieces were broken and disintegrated along the interface, which was found to happen slightly more in the concrete topping than the concrete base. This is because the roughness of the concrete base held the adhesive better than the concrete topping. The cohesive failure occurred when the applied horizontal load exceeded the bond strength at the concrete interface.

## Numerical analysis

### Interface shear stress distribution analysis

**Fig 11:**
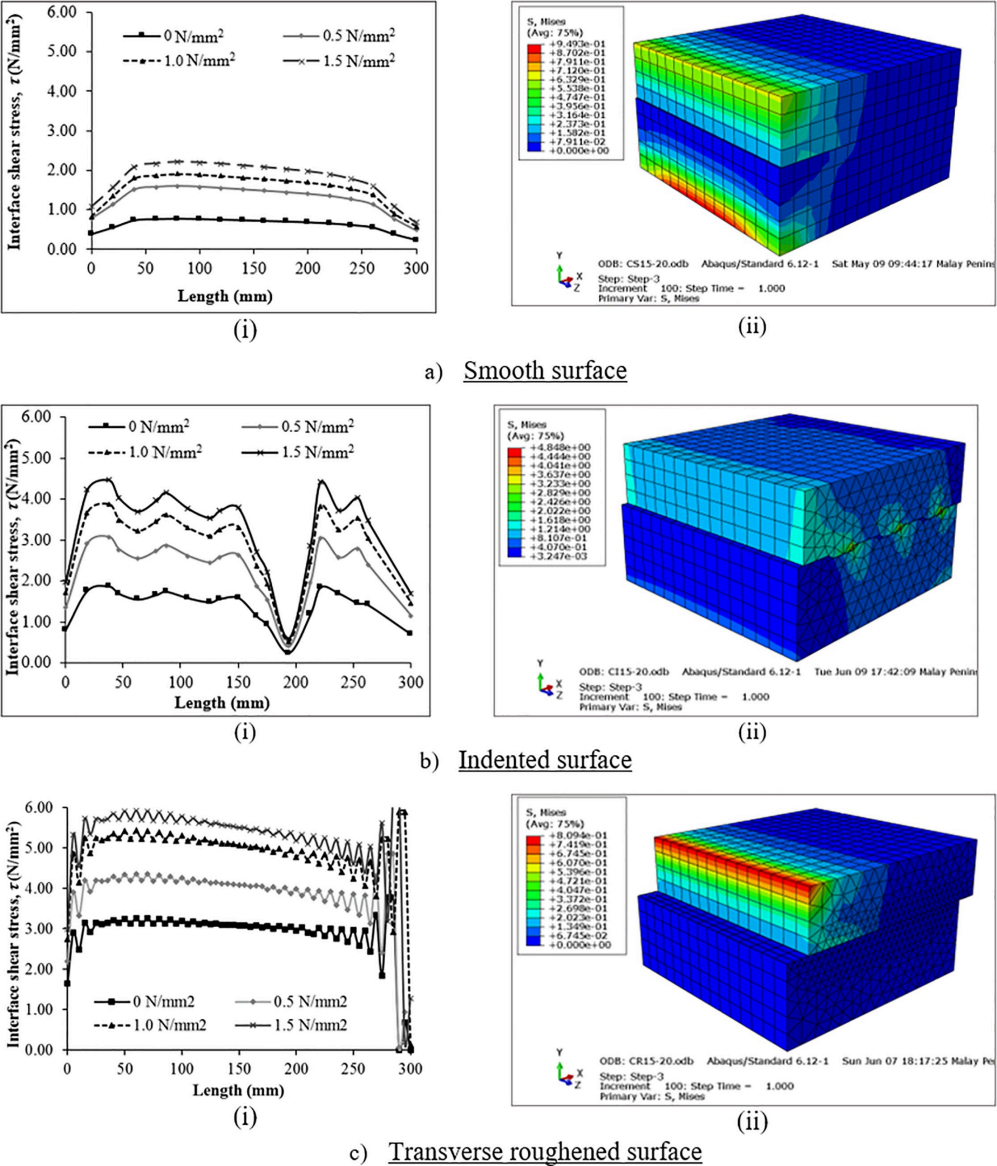
Interface shear stress (i) evolution relationship and (ii) distribution at peak shear load.

Fig 11 shows the interface shear stress evolution and distribution patterns of all models. In general, the stress patterns of all models are almost the same for all the applied normal stresses. The magnitudes of the shear elevate corresponding to the normal stress from 0 N/mm^2^ to 1.5 N/mm^2^.

In Fig 11(A), the interface shear stresses of the smooth surface model increase steadily until the 40 mm length. These stresses then start to decrease steadily as the length increases before a drop in magnitude is more pronounced beginning from 260 mm to 300 mm lengths. An inspection of the model output confirms that the maximum stress occurred at the edge of the vicinity of the crack tip nodes in front of the loading point as shown in the deformed shape in Fig 11(A). The higher stresses at these nodes inflicted the slipping between the two concrete layers. The crack was initiated at the side of higher stresses. Then, the crack propagated along with the interface once the stress started to decrease. A sudden failure occurred at the interface bond when the stress proceeded outside the cohesive zone to the fracture zone.

The interface of the indented model is found to have high stresses at the edge of the composite concrete, which is also at the onset of the crack propagation, as demonstrated in Fig 11(B). Although the undulations of the surface texture restrained the two concretes from a total separation, the high stress developed at the undulations between the concrete topping and concrete base caused the slipping of the interface as shown in the deformed shape in Fig 11(B). The stress was found to be increasing until the 37.5 mm length. This is likely to be the critical region for the indented surface before the strength reduction occurred. The stress started to decrease and increased alternately in a downturn manner under all the considered applied normal stresses until the 150 mm length. The fluctuation of the interface shear stress was likely to occur in such a fashion due to the restraint offered by the indented morphology along with the interface. As the load was increased, a major shear crack was developed and extended from the loading point to the 150 mm to 193 mm lengths. This caused a sharp drop in the interface shear stress. However, once the system was re-stabilized, the interface continued to resist the shear as the stress once again rose sharply until the point of 221.36 mm length, which is at the highest stress for all considered normal stresses. The strength at the interface began to weaken as the stress decreased until it made a sharp drop between the 262.50 mm and 300 mm lengths to complete the crack propagation along the interface.

Fig 11(C) exhibits that the interface shear stresses of the transversely roughened model increase consistently from the point of loading until the 5 mm length before decreasing at the 10 mm length. At the 35 mm length, stresses decreased slightly and became almost constant before rising between the 275 mm and 300 mm lengths. The highest stress was observed at 285 mm length due to the roughened surface resistance against the completion of the crack propagation at the interface. The interface layer in the concrete topping broke apart at this stage whereby at the end of the crack tip, the roughened surface became distorted. This is supported by the simulation of the cohesive failure mode of the interface based on the assumption that a small part of the concrete topping was cut-off close to the interface. Then, the sudden drop in shear was observed at the end of the 300 mm length. Since the surface was roughened, the fluctuation of high and low stresses depended on the irregularities of the surface in the FE model.

In general, the maximum stresses in all models occurred at the edge of the vicinity of the crack tip nodes in front of the loading point at 10% of the length along the interface leading to a slippage between the two concrete layers. Then, the crack propagated along the interface once the stress began to decrease gradually due to the restraint provided by the surface texture roughness. Then, a sudden failure occurred in the critical region when the stress went outside the cohesive zone to the fracture zone at around 90% of the length along the interface.

## Conclusions

The ‘push-off’ integrated with the normal stress testing method was employed to study the interface shear properties of the bonding of concrete base and topping. The composite concrete was designed such that the shear failure was the dominating mode. For a detailed understanding of the shear stress formation, FE simulation was also conducted.

Based on the findings obtained from the research work, the following conclusions can be drawn:

- The failure shear load increased corresponding to the rise in the normal stress from 0 N/mm^2^ to 1.5 N/mm^2^.
- The roughness of the top surface of the concrete base influenced greatly the interface shear strength of the concrete-to-concrete bond, from which the transversely roughened specimen exhibited the highest strength as compared with those with smooth and indented interface with the ranking: Transversely roughened > indented > smooth.
- A bi-linear relationship was observed in the horizontal load-interface slip curve for all interface textures.
- The smooth and indented surface textures failed by the adhesive failure mode where a thin slice of the concrete topping adhesive was observed on the top surface of the concrete base. However, the transversely roughened surface was dominated by the cohesive failure mode, in which the concrete chipping was observed on the top surface of the concrete base at failure.
- The crack tip edges suffered the highest stress caused by the increase of the interface shear stress during loading, which resulted in the breakage of the interface bond.
- Differences in the interface shear strengths between the FE models and experimental test specimens were 6% to 29%, 4% to 14%, and 3% to 21% for the smooth, indented, and transversely roughened surfaces, respectively, exhibiting good agreement between the two approaches.
